# Should LLMs be over empowered for high-stake regulatory research?

**DOI:** 10.1093/bib/bbaf299

**Published:** 2025-06-27

**Authors:** Partha Pratim Ray

**Affiliations:** Department of Computer Applications, Sikkim University, Gangtok 737102, Sikkim, India

**Keywords:** LLM, regulatory framework, shot learning, NLP, responsible AI

## Abstract

This letter critically evaluates the feasibility of implementing open-source large language models in regulatory research, building upon the recent study on zero-shot and few-shot learning approaches for regulatory tasks. While the study demonstrates that models like Flan-T5 can effectively extract pharmacokinetic drug–drug interactions and intrinsic factors from Food and Drug Administration (US) drug labels with high precision, it also highlights significant challenges, including computational constraints, performance variability, prompt sensitivity, and the risk of misclassification. To address these issues, this letter discusses intuitive ways for mitigating these limitations.

I write in response to the recent article “Harnessing large language models’ zero-shot and few-shot learning capabilities for regulatory research” published in Briefings in Bioinformatics [1]. The study offers a thorough evaluation of open-source large language models (LLMs), namely Flan-T5, Tk-Instruct, T0pp, and Vicuna, implemented within a secure, local regulatory network. By comparing these models against a baseline BioBERT, the authors demonstrate that, when applied in a zero-shot setting, these LLMs can achieve performance metrics comparable to or even exceeding those of models fine-tuned on extensive datasets. For instance, Flan-T5 achieved around 80% precision in identifying pharmacokinetic drug–drug interaction (PK–DDI) sentences and extracting intrinsic factors affecting drug exposure from Food and Drug Administration (FDA) labels without task-specific training, underscoring the transformative potential of zero-shot learning, particularly in contexts where curated training data are limited or sensitive.

The findings are both encouraging and thought-provoking. In regulatory research, especially in drug safety and pharmacovigilance, the rapid analysis of vast quantities of complex, domain-specific text is crucial. Deploying a zero-shot LLM within a secure local network can mitigate data breach risks commonly associated with online model usage and reduce the substantial costs related to creating large, labeled datasets. The study’s demonstration of processing over 700,000 FDA label sentences to extract clinically relevant data illustrates how such models can enhance operational efficiency and data privacy within regulatory agencies.

A critical strength of the study is its focus on prompt engineering. The authors show that providing prompts enriched with technical definitions, such as those for “pharmacokinetics” and “intrinsic factors”, significantly improves model performance. This insight is essential for regulatory applications where the accurate interpretation of drug label language depends on subtle contextual cues. However, the study also identifies key challenges. One major concern is the computational resource requirement; due to hardware constraints, the research was limited to models with up to 20 billion parameters, while more advanced open-source LLMs can have three to five times more parameters. This raises questions about the scalability of the observed performance benefits without corresponding infrastructure investments.

Variability in few-shot learning scenarios presents another challenge. Although Flan-T5 performed robustly in both zero-shot and few-shot settings, models like T0pp and Vicuna displayed inconsistent results with minimal training examples. This variability highlights the need for careful prompt tuning and, in some cases, additional training data to ensure consistent accuracy across diverse regulatory texts. Furthermore, error analysis reveals that even state-of-the-art LLMs may misclassify sentences due to ambiguous phrasing or insufficient context, such as misidentifying neutral sentences as positive for intrinsic factors, emphasizing the importance of human oversight in regulatory decision-making.

The broader implications of integrating LLMs into regulatory frameworks are significant. Deploying these models within secure local networks offers clear advantages in data privacy, cost efficiency, and operational scalability. Yet, given the risk-averse nature of the regulatory field, where public health and safety are paramount, any transition toward automated NLP tools must be accompanied by robust validation protocols and clear guidelines for human-machine collaboration. For instance, the study’s suggestion of a mixture of experts (MoE) strategy, which dynamically routes tasks to specialized sub-models, offers a promising avenue for mitigating current limitations and ensuring the adaptability and reliability of LLM outputs in complex regulatory contexts [2, 3].

As a final thought, the research presents a compelling case for the use of open-source LLMs in regulatory research, particularly through zero-shot learning. The rigorous experimental design and detailed performance evaluation provide strong evidence that these models can substantially reduce the need for extensive training data while maintaining high accuracy. The study judiciously addresses challenges such as computational constraints, variability in model performance, and prompt sensitivity, and it outlines practical pathways for future improvement, ranging from enhanced prompt engineering to hybrid ensemble strategies [4]. While the implementation of these technologies in a regulatory setting is not without its challenges, the potential benefits in data security, operational efficiency, and rapid adaptability make further exploration of LLMs in this domain both promising and necessary [5]. By embracing robust, secure, and contextually accurate NLP tools, regulatory agencies can significantly enhance their capacity to safeguard public health while leveraging the advances of artificial intelligence in high-stakes decision-making environments.
